# How basic public health services shape social integration: Evidence from domestic migrants in China

**DOI:** 10.1371/journal.pone.0340634

**Published:** 2026-02-03

**Authors:** Jiahao Zhao, Xinyi Zhu, Zhisen Gu, Sining Zheng, Zixiao Jiang

**Affiliations:** 1 School of Public Administration and Law, Fujian Agriculture and Forestry University, Fuzhou, China; 2 Faculty of Health and Life Sciences, INTI International University, Nilai, Malaysia; Shanghai Institute for Biomedical and Pharmaceutical Technologies, CHINA

## Abstract

The demand for and awareness of utilizing resident health services are increasing, yet uneven development across regions has led to uneven population mobility. Against this backdrop, as a crucial safeguard for the healthy lives of the mobile population, basic public health services profoundly influence the quality of life and development opportunities of these populations in urban settings. To elucidate the impact of basic public health services on the social integration of the urban mobile population, this study utilizes data from the China Migrants Dynamic Survey and employs empirical analysis to investigate the multidimensional effects of basic public health services on the social integration of domestic migrants. According to the results of the study, (1) basic public health services contribute to enhancing the social integration of domestic migrant population. This conclusion remains valid after robustness checks, with self-assessment of health playing a mediating role, and (2) the impact of basic public health services on the social integration of the domestic migrant population varies across different urban groups. This study contributes to examining and clarifying the policy effects of basic public health services in promoting the social integration of domestic migrants, providing empirical evidence for using basic public health services as a key lever to facilitate the social integration of domestic migrants.

## 1. Introduction

In 2019, the United Nations introduced the goal of “Universal Health Coverage (UHC),” urging all member states to strive toward achieving UHC by 2030 and setting clear requirements for health coverage. Following the global experience of the COVID-19 pandemic, a major public health event, public awareness and demand for health services have significantly increased. This has also raised greater expectations for the inclusivity, accessibility, and equity of basic public health service systems worldwide. During the pandemic response, many countries revealed inadequacies in preparedness and coverage of health services for mobile populations [1], highlighting the urgency and practical necessity of improving basic public health service systems. As a vital component of essential public services, basic public health services form a fundamental safeguard for the health of mobile populations. In modern society, where population mobility is increasingly frequent, ensuring that every citizen, regardless of migration status, can access continuous and convenient health services has become a critical and universally relevant challenge.

In China, the rapid advancement of urbanization has led to a sustained expansion of the mobile population. The country’s unique dual urban-rural household registration system differs significantly from Western models of residency management [2]. Constrained by this dual-structure framework, mobile populations have long been unable to enjoy the same rights and benefits as local residents, which has, to some extent, slowed their process of social integration. As a multidimensional concept, social integration refers to the process through which migrant populations assimilate into host societies across economic, behavioral, psychological, and cultural dimensions [3]. The level of integration achieved directly influences not only the long-term development and well-being of mobile populations in cities but also the overall stability and harmony of society.

In contrast to the steady advancement of urban development, the role and influence of public health services in facilitating the integration of domestic migrants into host communities remain underexplored. Against this backdrop, this study constructs a model grounded in relevant theories and employs data from the CMDS2017 (the China Migrants Dynamic Survey)—a nationwide survey executed by the China Population and Development Research Center using a stratified, multi-stage, probability-proportional-to-size (PPS) sampling method, covering 31 provinces (including autonomous regions and municipalities) and the Xinjiang Production and Construction Corps, and released in November 2017-- to conduct an in-depth analysis of the impact of basic public health services on the social integration of the domestic migrants. It further examines the variations in this impact across different social integration subgroups. The findings serve to validate and extend both social integration theory and push-pull theory. Finally, the paper offers corresponding policy insights. This research not only contributes to understanding the role of basic public health services in improving the social integration of domestic migrants but also deepens the recognition of the importance of equalizing access to such services. It thereby provides empirical evidence to support further efforts in promoting the equitable provision of basic public health services.

## 2. Literature review

In 1997, the World Health Organization expanded the service content to the functional framework of basic health services, laying the foundation for the development of a framework of modern basic public health services.

Research on the influencing factors of these domestic migrants‘ utilization of public health services rests primarily on empirical analysis. Grossman and other scholars discussed many factors affecting the floating population’s use of public health services, among which income was their common conclusion [4–7]. Other factors influence the utilization of public health services, and Hanson divided them into dimensions, such as population, family, community, and public health services [8].

In China, With the policy of public service health system construction approaching perfection, academic research is increasing. Research on the difference of public health service supply is a major research direction. Currently, research on basic public health services primarily focuses on the issue of “equalization.” Existing studies indicate that the provision level of basic public health services in China varies significantly across regions, with notable disparities observed between the eastern, domestic migrants central, and western regions, between the northern and southern parts, and between urban and rural areas [9–11]. Some studies take densely populated areas as pilot research areas to analyze the factors that affect the domestic migrants active access to basic public health services in cities, including age, whether they enjoy medical security, salary level, and self-rated health degree [12–16].

The theory of social integration, originating in Europe, aims to resolve differences between social groups. Internationally, the study of social integration theory is internalized into the study of measurement dimension. Milton Gordon, who proposed the early framework of the dimension of social integration, constructed a research framework for the synergy between institutions and culture. On this basis, Godras et al. used a multivariate model of migrants’ social adaptation to enrich the research system and selected the index system of social integration to cover economic status, social culture, social adaptation, political participation, and identity. In China, scholars have employed this theory to examine how migrants integrate into the local society. They have proposed a novel model that encompasses four aspects: economic, cultural, behavioral, and identitarian [17,18], upon which they have developed a range of evaluation dimensions, including employment, housing, environment, income, and consumption [19,20]. In addition, Katrine’s research specifically attributes this inclusion to various factors, such as work opportunities and education policies [21]. This study categorizes social integration into four facets: economic foothold, social adaptation, cultural integration, and psychological identity.

Research on the relationship between social integration and health services indicates that enhancing social integration can not only facilitate the establishment of health records [22] but also advance the equalization of healthcare services by strengthening economic sustainability, individual participation, and resource utilization efficiency [23]. Although representative studies on correlation between social integration and basic public health services are relatively few, social integration has been found to play a crucial role in the progress and development of health undertakings.

(1) Current research on the domestic migrants and public health services is extensive. However, studies on regional and group differences are lacking. Thus, this study takes the data of different regions of the country as an example and conducts research by groups to explore fully the impact of basic public health services on the social integration of different groups of the urban domestic migrants.
(2) Most existing studies separate public health service and social integration. Although a few analyses mention the impact of public health service on social integration, they lack the support of actual cases and data. Therefore, this study uses CMDS to determine the impact of the supply and access of basic health services on the social integration of the domestic migrants, as well as the differences among different groups, to provide targeted suggestions for the provision of basic public health services for different groups.

## 3. Theoretical foundations and research hypotheses

Given the rapid urbanization in China, Gu and Jian proposed the push–pull theory, which aims to explain how rural labor is attracted to urban areas due to pulling forces. This theory emphasizes the pulling forces at the place of relocation [24].

This study suggests that basic public health services, as a factor attracting rural populations to cities, play a significant role in promoting population migration and aggregation. However, during the process of labor transfer, supply-side issues, such as inequality and insufficiency, exist with these services. Therefore, the study proposes the need to eliminate these supply barriers to enhance the social integration of the domestic migrants. Simultaneously, the push factors in the areas of origin must be addressed to mitigate their negative impacts and collaborate with the destination areas to strengthen the monitoring of the mobile population.

The push–pull theory underscores the critical role that essential public health services play in the urban existence of migrant communities. Academic research also indicates that efficient provision and protection serve as significant pull factors for stabilizing life. A robust system is indispensable in addressing the vulnerability of the domestic migrants and elevating their level of integration [25,26]. In January 2021, The General Office of the Central Committee of the Communist Party of China (CPC) and the State Council of China issued the Action Program for Building a High-standard Market System, which envisions the pilot implementation of a hukou registration system based on permanent residency, aiming to eliminate disparities in hukou registrations and facilitate the social integration of domestic migrants.

This analysis leads to the formulation of hypothesis **H1: Basic public health services significantly affect the social integration of the domestic migrants, and the more services supplied and accessed, the higher the level of social integration.**

Regarding the role of health in the social integration of the domestic migrants, health serves as a fundamental basis for their urban life and development. Good health not only contributes to enhancing their personal quality of life and work efficiency but also creates conditions for their more comprehensive participation in economic and social activities. This, in turn, may facilitate their social integration across multiple dimensions, including economic, social, cultural, and psychological aspects. Consequently, this paper proposes hypothesis **H2: Basic public health services enhance the level of social integration among the domestic migrants by improving their health status.**

According to the New Economic Geography model, human migration—driven by economic factors such as wage levels and employment opportunities—flows from “peripheral” cities to central cities [27]. The new generation of mobile groups exhibits greater concern for the quality of life, surpassing the older generation in terms of social security, quality, and vocational ability. As they establish a foothold in the labor market, they gain access to superior-quality services and integrate more seamlessly into the local culture, and social participation and identity increase significantly. Consequently, this paper proposes hypothesis **H3: Compared with the older generation of migrant workers, basic public health services exert a more pronounced influence on the social integration of those under the age of 60.**

China’s traditional household registration system has been a barrier to the social integration of the domestic migrants. This system has also facilitated their gradual adaptation to local customs and lifestyles by improving their health and economic standing, which in turn enables deeper and more sustained social participation—the primary pathway to cultural adaptation [26]. Likewise, when it comes to mobility patterns, intra-provincial migrants are less affected. Consequently, this paper proposes hypothesis **H4: Compared to urban-hukou domestic migrants, basic public health services have a more significant impact on the social integration of rural-hukou migrants; compared to intra-provincial migrants, these services exert a stronger influence on the social integration of inter-provincial migrants.**

In addition, the female domestic migrants, especially the new generation of female domestic migrants, are subjected to the dual pressure of childbearing and employment discrimination and have a stronger demand for public health services; moreover, their social integration is significantly affected by the supply and access to basic public health services. Consequently, this paper proposes hypothesis **H5: Compared to males, basic public health services exert a more pronounced effect on the social integration of female migrants.**

**Fig 1:**
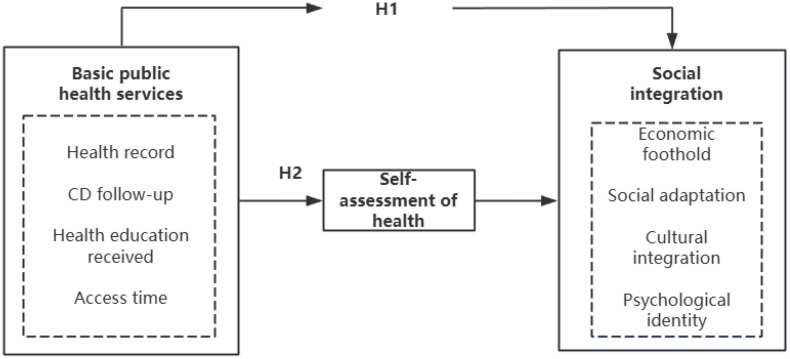
Theoretical framework diagram.

The theoretical framework for this analysis is depicted in Fig 1.

## 4. Data sources and indicator construction

### 4.1. Data sources

This study used data from the CMDS2017. The dataset covers 31 provinces (autonomous regions and municipalities) and the Xinjiang Production and Construction Corps across the country. This paper references the definition of the domestic migrants from the questionnaire and, in conjunction with the specific research items, selects the following criteria: “male and female domestic migrants aged 15 years and above (born in April 2002 and before) who have been living in the local area for six month or more and who do not have a household registration in the district (county or city).”

### 4.2. Construction of a social integration indicator system for domestic migrants

In selecting the article database and formulating the question set, we adhere to the concept of social integration for these sizable domestic migrants; thus, this study through the existing question dimensions in the CMDS database. constructed with **economic foothold, social adaptation, cultural integration, psychological identity** of the four subdimension indicators and **social integration** of a general dimension indicator. The economic foothold, measured by the dimensions of Jing and Sheng, refers to the degree of integration into the local living economy and consists of three main subdimensions: type of housing, social security status, and work status [28]. Social adaptation refers to the perceived level of participation in life. Cultural integration is mainly divided into two sub-dimensions: cultural habits and environmental acceptance. Psychological identity is divided into two dimensions: willingness to resid and identity recognition. The questionnaire comprises 21 distinct questions under each subdimension, which are designed to be mutually exclusive and inherently logical. Due to space limitations, the assignment table for the various dimensions of social integration will be provided as supplementary material.

#### 4.2.1. Secondary indicators: Economic foothold, social adaptation, cultural integration, psychological identity.

With the economic foothold indicators of the mobile population taken as an example and on the basis of their indicator construction, this study selected six questions to measure, utilizing factor analysis (Table 1) to extract common factors. Two effective factors were identified and then used to form a comprehensive score. Given that the comprehensive score is relatively abstract, the method proposed by Hu for processing such scores was applied, ultimately yielding a comprehensive score ranging from 1 to 100 [29]. Currently, the degree of economic foothold for the mobile population stands at 47.565, which is below the medium level. Similarly, the degree of social adaptation for the mobile population is calculated to be 8.024, indicating a relatively low level of participation in various social activities. The degree of cultural integration for the mobile population is 72.350, differing significantly from the previous two indicators, suggesting that the level of cultural integration among the mobile population is relatively high. The psychological identification level of the mobile population is recorded at 73.392, and the level of psychological integration among urban mobile populations also remains at a relatively high level.

**Table 1:** Statistics for various indicators of economic foothold for urban mobile population.

Item	Maximum	Minimum	Mean	Standard deviation	Factor loading 1	Factor loading 2
Self-Owned Housing	1	0	0.299	0.454	0.343	−0.423
Social Insurance Participation	3	0	1.011	0.345	0.6920	0.1162
SSCard Holder	1	0	0.458	0.498	−0.7494	0.0074
Labor contract signed	1	0	0.562	0.496	0.3925	0.5744
Job Search Difficulty Change	12.206	3.912	8.012	0.695	0.1124	−0.6548
Cumulative variance contribution rate (%)	59.97%					
KMO value	0.72					
Economic foothold	100	1	47.565	16.620		

#### 4.2.2. Overall indicator: Social integration.

To more accurately study the characteristics of social integration, this article drew on the practices of previous research by conducting a comprehensive analysis of the statistical results of all dimensions and referring to the previous sub-dimensions [30]. Given the numerous issues involved, exploratory factor analysis was adopted, and the KMO value of each dimension in Stata13 reached 0.777; with reference to the results, a total of six common factors with an eigenvalue greater than 1 were extracted, and the cumulative variance contribution rate was 50.91%, which conformed to the conditions of the factor analysis. These results were similarly converted to values ranging from 1 to 100. The statistical results showed that the mean value for social integration was 36.821, indicating that the overall level of social integration is still relatively low. This finding aligns with prior research on the social integration level of the domestic migrants, possessing realistic objectivity.

## 5. Empirical analysis

### 5.1. Variable selection

The selection of variables is sourced from CMDS2017.

#### Explained variable.

Given that social integration is a composite concept, in the previous analysis, it was divided into four sub-dimensional concepts, i.e., economic foothold, social adaptation, cultural intermingling, and psychological identity, as well as the total dimension of social integration for ease of study.

#### Core explanatory variable.

This study divided basic public health services into two dimensions, i.e., supply and access for research [26] to explore the relationship between the two factors and the social integration of the domestic migrants. Among them, supply refers to the services provided by the provider, i.e., whether to establish a health record or whether to follow up chronic diseases. Access refers to services accessible to the domestic migrants, i.e., number of individuals who have received health education and the time to the nearest basic public service point.

#### Control variables.

The selection of control variables in this article primarily refers to the current research in the field of demography concerning the domestic migrants, and is mainly divided into four factors related to demographic characteristics, health characteristics, mobility characteristics, and social network, in line with the analysis of social integration of the characteristics of the domestic migrants. The control variables have been tested for multicollinearity, and the resulting VIF value is 1.12 (<5), indicating that no issue of multicollinearity exists, and the selection of control variables is appropriate. The processing and assignment of variables are shown in Table 2.

**Table 2:** Definition and descriptive statistics of model variables.

Primary indicator	Secondary indicator	Definition and assignment of variables	Mean	Std. Dev.	Max	Min
**Explained variables**						
Economic foothold		Value Range 1–100	47.565	16.620	1	100
Social adaptation		Value Range 1–100	8.024	10.613	1	100
Cultural integration		Value Range 1–100	72.350	17.748	1	100
Psychological identity		Value Range 1–100	73.392	17.748	1	100
Social integration		Value Range 1–100	36.821	14.506	1	100
**Core independent variable**						
Supply	Health record	Yes = 1; No = 0	0.308	0.462	0	1
	CD follow-up	Yes = 1; No = 0	0.350	0.477	0	1
Access	Health education received	0–9	3.542	3.376	0	9
	Access time	Within 1–15 minutes = 1; 15 minutes exclusive – 30 minutes inclusive = 2; 30 minutes exclusive – 1 hour inclusive = 3; Over 1 hour = 4	1.183	0.444	1	4
**Control variables**						
Demographic characteristics	Age	15–19 = 1; 20–39 = 2;40–59 = 3; > 59 = 4	2.885	0.510	1	4
	Gender	Male = 1; Female = 0	0.698	0.459	1	0
	Marital status	Married = 1; Unmarried = 0	0.936	0.247	1	0
	Educational level	No Formal Education = 1; Primary school = 2; Junior high = 3; High school/vocational school = 4; Junior college = 5; Bachelor’s degree = 6; Postgraduate degree = 7	2.053	0.941	1	7
	Party member	Party Member = 1; Non-Party Member = 0	0.071	0.256	0	1
	Household Registration status	Agricultural = 1; Non-Agricultural = 0	0.842	0.365	0	1
Health characteristics	Self-assessment of Health	Unhealthy, unable to take care of oneself = 1; Unhealthy, but can take care of oneself = 2; Basic health = 3; Healthy = 4.	3.4056	0.6743	1	4
Mobility characteristics	Length of Residence	Years of Residence in the Destination Area	10.010	7.550	1	51
	Mobility Scope	Within-city Migration = 0; Cross-province Migration = 1	0.509	0.500	0	1
Social network	Accompanying migrants	Number of Relatives in the Local Area for the Sample	3.226	1.097	1	10

### 5.2. Model construction

Given that the explanatory variable of this study, i.e., social integration, is a continuous variable ranging from 1 to 100 for various dimensions, the ordinary least squares model is selected for model construction, which is consistent with the characteristics of model construction. On the basis of the current available research, the model expression of this study is constructed as follows:


$$Yble=\alpha +\beta_{\text{1}}x_{\text{1}}+\beta_{\text{2}}x_{\text{2}}+\ldots +\beta_{n}x_{n}+\varepsilon$$

In the above equation, *ble* represents the degree of social integration of urban domestic migrants, including four subdimension indicators of economic foothold, social adaptation, cultural integration, psychological identity, and the total dimension of social integration; Among them, α represents the constant term, $\beta_{\text{1}}$ to $\beta_{n}$ are the regression coefficients for each variable, $x_{\text{1}}$ to $x_{n}$ are the core explanatory variables and control variables in this study, and ε is the random disturbance term.

### 5.3. Empirical analysis and result interpretation

#### 5.3.1. Analysis of basic regression results.

Table 3 presents the basic regression results of the model. On the basis of these results, we proceed to interpret the core explanatory variables.

**Table 3:** Regression results of the impact of basic public health services on the social integration of urban migrants.

Explained variable	Economic foothold	Social adaptation	Cultural integration	Psychological identity	Social integration
Health record	3.432** (1.369)	2.805*** (0.314)	2.891*** (0.477)	3.443*** (0.562)	4.400*** (1.143)
Health education received	0.209 (0.190)	0.604*** (0.043)	0.574*** (0.065)	0.674*** (0.077)	0.444*** (0.159)
CD follow-up	4.870*** (1.294)	2.399*** (0.304)	1.595*** (0.463)	3.147*** (0.544)	3.700*** (1.091)
Access time	0.380 (1.280)	−0.091 (0.329)	−1.088** (0.542)	−1.527*** (0.586)	−2.218** (1.072)
Age	−2.188* (1.124)	−1.462*** (0.302)	0.847* (0.459)	1.014* (0.540)	2.771*** (0.938)
Gender	5.050*** (1.279)	1.297*** (0.324)	0.024 (0.492)	−0.662 (0.580)	3.998** (1.068)
Marital status	−1.278 (2.226)	−0.850 (0.627)	0.549 (0.953)	−0.734 (1.123)	−0.862 (1.859)
Educational level	3.900*** (0.775)	2.365*** (0.186)	1.427*** (0.282)	1.290*** (0.333)	4.511*** (0.647)
Party member	7.026** (3.102)	8.780*** (0.598)	1.630* (0.909)	3.453*** (1.071)	8.596*** (2.590)
Household registration status	−1.455 (1.794)	−1.554*** (0.445)	−2.015*** (0.676)	−3.313*** (0.796)	3.592** (1.498)
Self-assessment of Health	5.450*** (1.864)	−0.244 (0.472)	0.439 (0.717)	−0.700 (0.845)	3.266* (1.557)
Length of residence	−0.225*** (0.082)	0.033* (0.019)	0.155*** (0.030)	0.297*** (0.035)	0.101 (0.068)
Mobility range	5.545*** (1.220)	−0.058 (0.290)	−1.658*** (0.441)	−2.620*** (0.520)	1.260 (1.019)
Accompanying migrants	1.512*** (0.575)	0.238* (0.141)	0.067 (0.215)	0.123 (0.253)	1.331*** (0.480)
Constant	33.246*** (5.239)	6.248*** (1.402)	65.815*** (2.131)	69.038*** (2.511)	30.807*** (4.614)
$R^{\text{2}}$	0.223	0.172	0.036	0.050	0.263
N	4506	4506	4506	4506	4506

Note: *, **, *** represent significance at the 10%, 5%, and 1% levels, respectively. Robust standard errors are provided in parentheses. The same applies below.

(1) **Whether to establish health records.** Establishing a health record has a significant impact on social integration [31]. Except for economic stability, which is significant at the 5% level, all other dimensions are significant at the 1% level. Establishing a health record can reduce unnecessary medical time waste and improve the quality of medical care, thereby enabling individuals to better engage in work and assist the domestic migrants in stabilizing their economic foothold. In terms of social adaptation, the domestic migrants who establishes health records can more accurately assess the service, thereby identifying issues in social governance and enhancing their level of social participation and monitoring capabilities. In terms of cultural integration, establishing health records facilitates contact between health record agencies and registered urban domestic migrants to provide targeted services, promoting their acceptance of local culture [32]. As for psychological identification, health records can positively impact the psychological identification of domestic migrants because they signify the importance placed on this group by the government and community, strengthening their psychological identification.
(2) **Level of health education received.** The level of health education directly affects social adaptation, cultural integration, and psychological identification and is significant at the 1% level. Through health education, the domestic migrants can better understand how to maintain health and related policies. This makes them more concerned not only about their own health but also about their relatives and social circles [33]. Furthermore, health education can help domestic migrants better understand the hygiene habits of the local population, leading to a significant improvement in cultural habits and environmental acceptance. In terms of psychological integration, more health education prompts domestic migrants to have a certain degree of identification with the local population, resulting in significant improvements in their willingness to reside and their identity recognition.
(3) **Whether one has enjoyed chronic disease follow-up services**. The impact process of chronic follow-up services and the establishment of health records on social integration is similar, with a more direct effect on the dimensions of social integration. Follow-up services can effectively monitor the physical health level of domestic migrants, promote their health status, and facilitate their ability to work better, leading to improved economic stability. With a healthy physique, they are more willing to participate in the management of social affairs. Their specialized health monitoring leads to strong psychological identification, good social adaptation, and great willingness to absorb local culture. Overall, the level of social integration among domestic migrants increases due to their receipt of chronic disease follow-up services.
(4) **Access time for basic public health services.** The access time for basic public health services has a negative and significant impact on cultural integration and psychological identification at the 1% level but has no significant relationship with economic stability and cultural adaptation. As the distance between the basic public health service points and individuals’ homes increases, the longer it takes for domestic migrants to access public health services and the lower their cultural habits and environmental acceptance are, thus demonstrating a negative significant impact relationship. The impact on psychological identity can be summarized as follows: long-term migration makes the domestic migrants vulnerable and requires great support from public health services. If individuals experience significant delays in accessing these services, they may perceive a shortage of local healthcare, which could lead to a decrease in their desire to live in the area and a diminution in their sense of belonging. In summary, the previous hypothesis that basic public health services have an impact on some dimensions of social integration is partially supported by the analysis.

To verify the robustness of the regression results, this study employed the Poisson regression model for examination. Initially, a likelihood ratio test was conducted, and the results showed that the constrained model had a lower AIC compared with the unconstrained model, indicating that both models passed the test. As shown in Table 4, the regression results across various dimensions remained largely unchanged, suggesting that the regression results are robust.

**Table 4:** Robustness test results.

Explained variable	Economic foothold	Social adaptation	Cultural integration	Psychological identity	Social integration
Health record	0.069*** (0.013)	0.328*** (0.011)	0.040*** (0.004)	0.046*** (0.004)	0.113*** (0.015)
Health education received	0.004** (0.002)	0.071*** (0.002)	0.008*** (0.001)	0.009*** (0.001)	0.011*** (0.002)
CD follow-up	0.098*** (0.013)	0.289*** (0.011)	0.022*** (0.004)	0.042*** (0.004)	0.097*** (0.014)
Access time	0.007 (0.013)	−0.016 (0.012)	−0.018*** (0.004)	−0.021*** (0.004)	−0.062*** (0.015)
Control variable	Controlled				

#### 5.3.2. Mechanism analysis.

**Fig 2:**
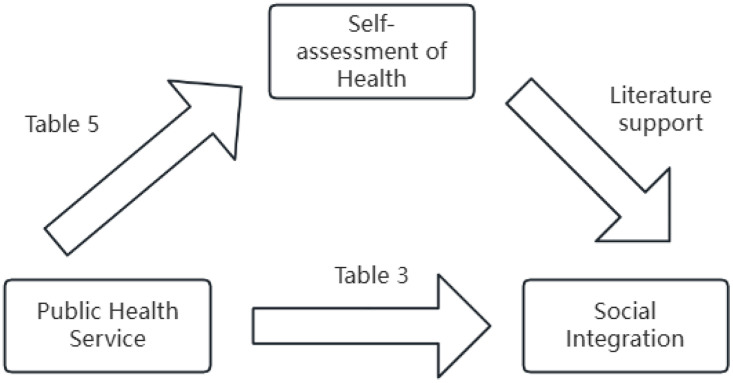
Influence mechanism analysis.


The basic regression analysis in table 3 reveals that basic public health services exert a substantial influence on the overall dimension of social integration. In this section, we adopt Jiang T’s two-step method for examining mechanism effects [34], introducing the concept of self-assessment of health. By analyzing the interaction between the supply and access of basic public health services (core explanatory variable) and self-assessment of health, we delve into the mediating role of health level in shaping social integration. The theoretical framework for this analysis is depicted in Fig 2.

$$Me=\alpha_{\text{0}}+bx+\alpha_{\text{2}}*controls+\varepsilon$$

Model 1 to Model 4 present the regression results of various dimensions of basic public health services and self-assessment of health. The regression results from Table 5 indicate that, within the realm of basic public health services, services other than the quantity of health education significantly enhance the self-assessment of health of urban domestic migrants, subsequently elevating their level of social integration. The reasons for this are as follows: 1) Health records enable health service providers to promptly grasp the health information of community-based domestic migrants and facilitate tracking, laying the groundwork for future public health service provision. 2) The effectiveness of health education may be limited by the degree of match between content, form, frequency, and patient needs, as well as the acceptability and engagement of patients. 3) Chronic disease follow-up services alleviate health concerns among the mobile young and middle-aged populations, fostering their social participation. 4) The longer the duration of access to health services, the more challenging it is to obtain them, thereby affecting self-assessment of health

**Table 5:** Regression results of the impact of basic public health services on the self-assessment of health.

	Self-assessment of Health			
	Model1	Model2	Model3	Model4
Health records	0.044** (2.06)	—	—	—
CD follow-up services		0.080*** (3.85)	—	—
Health education	—		0.001 (0.43)	—
Access time	—	—	—	−0.069*** (−3.07)
Control variable	Controlled	Controlled	Controlled	Controlled
Constant	3.642*** (39.52)	3.650*** (39.42)	3.638*** (39.58)	3.737*** (39.07)
$R^{\text{2}}$	0.032	0.031	0.034	0.033
N	4506	4506	4506	4506

From the perspective of health effects, international migration experiences show that individuals with better health are more willing and able to migrate. The healthy immigrant effect indicates that migrants are initially healthier than the local population, but over time, this health advantage diminishes and may even become worse than that of the locals. This phenomenon has been confirmed in many countries [35].

### 5.4. Heterogeneity analysis

Significant gaps are found in the impact of urban basic public health services on different groups. Given the detailed analysis of various dimensions in the previous sections, using urban integration indicators directly as a comprehensive measure to study group heterogeneity in this section is simpler and more intuitive. Models 5–12 present the empirical results of the impact of various basic public health services on social integration, as shown in Table 6.

**Table 6:** Heterogeneity analysis.

Explained variable	Mobility range		Gender		Age		Household registration	
	Model5	Model6	Model7	Model8	Model9	Model10	Model11	Model12
	Within-city Migration	Cross-province Migration	Male	Female	Age under 60	Age 60 and above	Nonagricultural	Agricultural
Health record	4.060*** (1.507)	2.343 (2.348)	4.451*** (1.455)	4.704** (1.816)	4.277*** (1.190)	4.978 (3.556)	3.923*** (1.222)	3.855 (2.972)
Health education received	0.730*** (0.232)	0.161 (0.217)	0.245 (0.207)	0.745*** (0.246)	0.451*** (0.165)	0.538 (0.551)	0.252 (0.170)	0.852** (0.414)
CD follow-up	6.182*** (1.581)	1.379 (1.491)	2.893** (1.379)	5.392*** (1.767)	4.012*** (1.150)	0.971 (3.152)	2.893** (1.379)	5.392*** (1.767)
Access time	−2.452 (1.706)	−0.650* (1.393)	−1.915 (1.418)	−2.256 (1.626)	−2.131* (1.117)	−4.848 (3.409)	−3.178*** (1.141)	−4.721 (2.870)
Control variable	Controlled							
$R^{\text{2}}$	0.275	0.374	0.130	0.509	0.310	0.108	0.276	0.397
N	2872	1634	1238	2464	1245	1605	1526	2103

From the perspective of group heterogeneity, the impact of health records on social integration varies significantly in terms of mobility range, age, and household registration. Social integration is more susceptible to the establishment of health records among those who migrate across provinces, are under 60 years old, and have nonagricultural household registration. The number of health education sessions has a more pronounced impact on social integration among those who migrate across provinces, are under 60 years old, and have agricultural household registration. The impact of chronic disease follow-up services on social integration is more evident among domestic migrants who migrate across provinces and are under 60 years old. The impact of basic public health service access time on social integration is more significant among those who migrate within the province, are under 60 years old, and have nonagricultural household registration. This finding also indicates that some assumptions of Hypotheses 3–5 are supported. Differences are found in the impact relationships between the utilization and access of various specific basic public health services.

## 6. Conclusions

On the basis of the CMDS2017, this study investigates whether basic public health services can affect the social integration of domestic migrants. The empirical results demonstrated that (1) basic public health services exerted a positive influence on various dimensions and the overall level of social integration among both domestic migrant groups. Moreover, health self-assessment played a more prominent mediating role in this impact. (2) After controlling for relevant factors, this study found that health record establishment, health education, and chronic disease follow-up services enhance the social integration of urban migrants, while the time taken to access public health services negatively affects their integration. (3) From the perspective of heterogeneity, the effect of basic public health services on social integration of female group is more significant than that of male group, and the effect on young and middle-aged group is more obvious than that of middle-aged and old people. Similarly, the cross-provincial domestic migrants is more strongly affected by basic public health services than the cross-city domestic migrants.

## 7. Discussion

To ensure basic public health services for domestic migrants, promote their social integration, and create a community environment that better suits their survival and development, the author proposes the following policy considerations: (1) Charmaine's research on the Canadian context has concluded that the equalization of basic public health services for the domestic migrants is a pivotal factor in enhancing their health status [36], To address this issue, the government should allocate more resources [37], broaden the range of services, streamline processes, establish health records, and integrate psychological support and health education to enhance their health consciousness. (2) We should enhance the establishment of a differentiated system for fundamental public health services. Thailand paid particular attention to the protection of vulnerable groups such as the poor, the elderly, children and persons with disabilities by providing them with free medical services [38]. Studies have shown that public health services have an impact on social integration, with different groups within the domestic migrants being affected in varying degrees calling for tailored approaches. The government should develop a differentiated public health service delivery system to cater to the diverse needs of these groups, especially women, cross-provincial migrants, and those with agricultural household registrations.

The academic contributions of this study are primarily reflected in two aspects. First, building on the push-pull theory and social integration theory, it constructs an indicator system for the social integration of the floating population, thereby extending the scope of these theoretical frameworks. Second, it connects public health services with the social integration of migrants, analyzing the impact of public health service provision on promoting such integration, as well as examining differences across social subgroups.

It should be noted that this study is based on data collected prior to the COVID-19 pandemic, after which the demand for public health services among domestic migrants has risen significantly. Additionally, as the research focuses on China’s domestic migrants, whether its conclusions are applicable to a broader international context remains to be further examined. Future research could expand the scope of inquiry—for example, by conducting cross-national comparative studies or taking the COVID-19 pandemic as a turning point to deeply analyze the evolving role of public health services among migrant groups. Through such research directions, we can better support the social integration of domestic migrants and contribute to the advancement of social equity and justice.
